# Characterization of the Growing From the Tip as Robot Locomotion Strategy

**DOI:** 10.3389/frobt.2019.00045

**Published:** 2019-06-20

**Authors:** Emanuela Del Dottore, Alessio Mondini, Ali Sadeghi, Barbara Mazzolai

**Affiliations:** Center for Micro-BioRobotics, Istituto Italiano di Tecnologia, Pontedera, Italy

**Keywords:** growing robot, kinematics, 3D navigation, bioinspiration, soft robotics

## Abstract

Growing robots are a new class of robots able to move in the environment exploiting a growing from the tip process (movement by growing). Thanks to this property, these robots are able to navigate 3D environments while negotiating confined spaces and large voids by adapting their body. During the exploration of the environment, the tip of the robot is able to move in any direction and can be kinematically considered as a non-holonomic mobile system. In this paper, we show the kinematics of robot growing at its tip level. We also present the affordable workspace analyzed by an evaluation of feasible trajectories toward target poses. The geometrical key parameters imposing constraints on growing robots' workspace are discussed, in view of facing different possible application scenarios. The proposed kinematics was applied to a plant-inspired growing robot moving in a 3D environment in simulation, obtaining ~2 cm error after 1 m of displacement. With appropriate parametrization, the proposed kinematic model is able to describe the motion from the tip in robots able to grow.

## Introduction

The ability of robots to move and interact with the environment is of fundamental importance for the accomplishment of demanded tasks in out-of-factory scenarios. Several kind of locomotion have been studied and adopted for different applications: in-pipe inspection (Mirats Tur and Garthwaite, 2010), medical (Phee et al., 1997; Dario and Mosse, 2003), aerial (Colomina and Molina, 2014), terrestrial (Siegwart et al., 2011), or marine (Seto, 2013) exploration. Among many solutions, animal-like locomotion strategies have been implemented in several different robotic platforms to improve performance and compliancy with the environment (Sfakiotakis et al., 1999; Armour et al., 2007; Bachmann et al., 2009; Cianchetti et al., 2015; Aguilar et al., 2016; Hooper and Büschges, 2017). More recently, plants have been explored in robotics leading to a new paradigm of locomotion, which is moving by growing (Sadeghi et al., 2013, 2014, 2017; Del Dottore et al., 2018b). This new class of robots can navigate the environment taking inspiration from the plants' feature to continuously increase the mass by adding new cellular material at their growing extremities, i.e., at shoot and root apexes (Verbelen et al., 2006). From an artificial perspective, this movement strategy can be exploited by additive manufacturing techniques (Sadeghi et al., 2017; Kayser et al., 2019) or skin eversion (Tsukagoshi et al., 2011; Sadeghi et al., 2013; Hawkes et al., 2017). This way, the robot is able to orient itself without the need of moving its entire body but confining the movement at its tip, while dynamically creating the robot's body and adapting its morphology to the environmental conditions and physical constraints. This feature qualifies robots able to grow for applications where the environment is not necessarily predefined or predictable, and high body adaptability is required (Laschi et al., 2016).

Being growth a new topic in robotics, the kinematics of such kind of movement is still poorly described in literature. Yet, to a certain extent, particularly from a kinematics point of view, a growing robot shows some similarities with systems implementing a follow-the-leader strategy, similarly to serpentine or hyper-redundant manipulators (Choset and Henning, 1999; Neumann and Burgner-Kahrs, 2016). This strategy of motion enables the extension of the backbone curve from the end effector location, while the antecedent part of the body follows the head direction. Such growth-like movement can be achieved either by propagating the curve forward from the base with the extension of discrete manipulator segments (Neumann and Burgner-Kahrs, 2016), and from the head down to the body (Choset and Henning, 1999), or with an extension from the tip with the release of a nested module (Gilbert et al., 2015; Kang et al., 2016). However, a follow-the-leader robot typically slides all its body, or a consistent part of it, during tip advancement, instead a growing robot permits to moves only its tip, while the consolidated structural body is fixed respect to the environment, reducing external friction and, thus, the energy required for moving (Sadeghi et al., 2013, 2014). Moreover, systems implementing follow-the-leader strategy are normally discretized, with a fixed number of segments (and joints) and a defined maximum length (and workspace in case of manipulators). On the contrary, a growing robot has not a predefined body, since this mainly depends by the added feeding material, and the robot can assume, in theory, an infinite number of configurations.

In the scenario of a robot growing at the tip and moving in space, the main question to be addressed is related to the path that the robot can take toward the target point, rather than the trajectory that the end effector makes to reach a specific point. In this view, we can compare the motion of this growing robot to that of a mobile robot able to navigate in a three dimensional space. To this end, it is important to describe the geometric configurations, or poses, of a growing robot and the potential environments that it can be able to navigate. Another important consideration is that a growing robot at the tip is a non-holonomic system, having a total of five DoF in configuration space (tip position in 3D space, and heading, and pitch angles) but only three controllable DoF at joint space, which are: two degrees of steerability (for tip orientation), plus one degree of mobility (the system velocity - in this case called growth velocity). These three degrees of maneuverability define together the space of possible configurations of a growing robot in 3D. For mobile robots, analyzing the workspace includes definition of how the robot moves between different poses, as well as of possible trajectories that the robot takes to reach a desired position with a specific orientation. The kinematic control of a system moving from a pose to another along a desired trajectory is often done by dividing the path in motion segments composed by straight lines and segments of a circle (Siegwart et al., 2011). When considering mechanical constraints, Dubin's path generation approaches (Dubins, 1957) are often used and adapted for the definition of feasible trajectories in 3D space (Ambrosino et al., 2009; Babaei and Mortazavi, 2010; Yu et al., 2015; Makdah et al., 2016).

In Del Dottore et al. (2018a), we provided a plant-inspired kinematic model, described in joint space, of a growing robot able to deposit new material from its tip in order to incrementally build its body (Sadeghi et al., 2017) and consequently move its exploratory tip forward. In that work we evaluated the error between target positions achieved in simulation and with the real robot after three different paths (2D curvilinear trajectories with arcs radius of 12.5, 17.5, and 22.5 cm), finding the maximal error of about 7% in 10 cm traveled in air. Here, we go forward providing a more thorough formalization of the kinematics, extending the description from joint to configuration space, and analyzing the space of maneuverability following the approach of non-holonomic systems. We present a strategy for defining suboptimal trajectories, with Dubin's path, for growing robots moving in 3D space and we describe the movements of the robot with our proposed kinematics. We also tested and evaluated our kinematic control in simulation parametrizing the model *in primis* with our plant-inspired growing robot, then testing robustness introducing different level of perturbations during growth, and finally with different settings of robot size and velocity.

In the following, we first describe the kinematics and the key design parameters affecting the behavior of growing robots (section Methods); then, we present the strategy proposed for defining 3D trajectories (section Results); and, finally, we discuss results of the simulations (section Discussions), followed by conclusive remarks (section Conclusions).

## Methods

### Kinematics of Growing Robots

The characterization of the motion of a robot requires the definition of its kinematics and strategy to move from a point A to a point B in its configuration space. Based on the Chasles' theorem, any robot starting from (point) A, with a certain orientation, can reach (point) B, with another orientation, by means of a translation followed by a rotation of the body about its initial position (Siciliano and Khatib, 2008).

**Figure 1:**
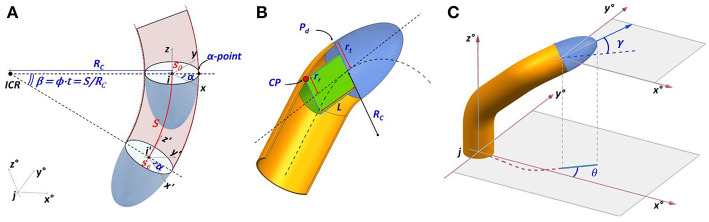
**(A)** Schematic representation for the motion from the tip of growing robots. Frame ***i*** is integral with the tip, which moves within an inertial frame ***j*·** A curvature with radius ***R***_***c***_ is induced by a greater material deposition over the deposition plane (***P***_***d***_) at a position identified with the angle **α**. After a period of time *t* have traveled a distance ***S*** of the arc around the center of rotation ***ICR***; **(B)** definition of pitch **γ** and heading **θ** angles; **(C)** visual overview of key parameters in the mechanics of growing robots with the contact point ***CP*** of internal components with the robot's body which define the minimum curvature radius.

In Del Dottore et al. (2018a) there is an introduction of the forward kinematics, inspired by plant growth, describing the motion from the tip in 3D space through homogeneous transformation matrices. Starting from that, we can generalize the formulation by describing *i* as a moving coordinate frame, integral with the robot's tip, and *j* as the inertial frame (Figure 1A) (the entire dictionary of the symbols used throughout the paper is available in Supplementary Material). The origin of coordinate frame *i* relative to coordinate frame *j* can be denoted by the 3 × 1 vector:

(1) pji=(xjiyjizji).

A generic point ^*i*^*r* ∈ ℝ^3^ in frame *i* can be expressed in frame *j* as ^*j*^*r* ∈ ℝ^3^ knowing the transformation matrix ^*j*^*R*_*i*_ ∈ *SO*(3), with the equation:

(2) rj=Rji ir+pji;

which can also be written as:

(3) (rj 1)=(Rjipji01×3   1)(ri1),

where the first factor of the right hand is the homogenous transformation matrix ^*j*^*T*_*i*_ ∈ *SO*(4). The forward kinematics of a growing robot can be described in the joint space, by identifying the joint-like position in the plane (P_*d*_ Figure 1B) between moving tip and body, where the process of growth is actuated. From a frame *i*, the next frame is obtained as a function of the growth velocity (*g*), position for the actuation of greatest material deposition [expressed as angle α w.r.t. the *x* axis in (frame) *i*] and intensity of bending in a unit of time (ϕ). With these actuation parameters we can formulate the sequence of post-multiplied transformations:

(4) Ti=Tz,αTtr,vTy,ϕTtr,−vTz,−α          = (Cα2Cϕ+Sα2CαSαCδ−CαSαCαSϕgCα(1−Cϕ)ϕCαCϕSα−CαSα Cα2+CϕSα2SαSϕgCα(1−Sϕ)ϕ−CαSϕ−SαSϕCϕgSϕϕ0001)          =(   Ripi01×3  1),

where the first subscript of *T* indicates if *T* is a translation (*tr*) or a rotation matrix (by indicating around which axis), and the second subscript gives the angle of rotation or direction of translation (*v*); and by convention: *C*_α_ = cos α and *S*_α_ = sin α. In (4), the greatest deposition is applied at the α-point along the circumference of the robot's tip, with respect to its *x-y* plane (Figure 1A) (the rotation *T*_*z*, α_ is used to localize this point and *T*_*z*, −α_ is used to rotate back the tip after the following transformations). Differently from Equation 6 in (Del Dottore et al., 2018a), the two discrete steps of motion (translation—for a vertical growth—and rotation—which describes a bending) are merged together in a single atomic action, obtained by *T*_*tr, v*_*T*_*y*, ϕ_*T*_*tr*, −*v*_, where *v* is the vector ${\left[{-R}_{c}\;0\;0\;1\right]}^{T}$, which is used to localize the inertial center of rotation (*ICR*), and then the rotation of ϕ about the *y* axis (Figure 1A). Since *R*_*c*_ can be expressed as relation between the intensity of bending and growth velocity ($R_{c}=\frac{g}{\phi }$), the matrix in (4) can be obtained by substitution. When no bending is applied (ϕ = 0), α = 0 and the last column will define a straight growth. The kinematic chain that describes the moves done (or to be done) by the tip (with frame *i*) from an initial configuration *s*_0_ to reach a final configuration *s*_*e*_ along its trajectory, in terms of frame *j*, is obtained by consecutive multiplication of the homogeneous transformation matrices obtained with Equation (4):

(5) Tji(se)=∏s0seTi(st).

As in mobile robotics, we can identify a path from *s*_0_ to *s*_*e*_ composed by a sequence of turns and straight lines (Dubins, 1957; Siegwart et al., 2011), and obtain the transformation matrix ^*i*^*T* (*s*_*t*_) for each of the segments. The problem is to define a feasible path for the robot.

To approach this problem, we describe the kinematics in configuration space for the tip of a growing robot with:

(6) $$\{\begin{array}{l} \;\dot{x}=g\cos\gamma \cos\theta \\ \;\dot{y}=g\cos\gamma \sin\theta \\ \;\dot{z}=g\sin\gamma \\ \;\dot{\theta }=u_{1}\\ \;\dot{\gamma }=u_{2}, \end{array}$$

where *g*, as previously defined, is the growth velocity, *x, y, z* are the components of ^*j*^*p*_*i*_, θ is the orientation of the tip in the *x-y* plane of frame *j*, or heading, and γ is the orientation of the tip with respect to the plane *x-y* in frame *j*, or pitch (Figure 1C). *u*_1_ and *u*_2_ are the control inputs, that need to be determined and should satisfy geometric constraints imposed by the robot mechanics on a minimum curvature radius reachable by the system (|*R*_*c*_| ≥ *R*_min_).

From a geometric point of view, the *R*_min_ is the main parameter limiting the affordable workspace of a growing robot, given a maximum allowable body displacement or that should be reached within a certain time. The minimum curvature radius is defined by geometric parameters of the mechanism with the following relations:

(7) $$\begin{array}{l} R_{\min}=\frac{L^{2}-{r_{t}}^{2}+{r_{r}}^{2}}{2\cdot \left(r_{t}-r_{r}\right)}, \end{array}$$

where *r*_*t*_, *r*_*r*_ and *L* are parameters dependent on robot design (Figure 1B). As in vehicles, *r*_*t*_ is the distance between the central line and the external lateral line where the wheel is located or, as in the case of growing robots, where the material is incrementally added; *L* is the distance between the steerable component (represented by the material added in the plane of growth actuation P_*d*_) and the backward extremity of non-steerable module, if any (ideally, the wheelbase in a vehicle); and *r*_*r*_ is the distance between the central line and the external side of the virtual cylinder encapsulating the internal components.

It should be noted that the parameters *r*_*r*_ and *L* in (7) strictly depend on the configuration of internal components, which can be arranged differently from a cylindrical shape; however, we can approximate the bulkiness with the virtual cylinder built around the most cumbersome component in the assembly, considering it symmetric respect to the central line.

*R*_min_ and the growth velocity *g* supply the maximum bending angle variation per unit of time [as in Equation (8)], which also represents the constraint for both control inputs (*u*_1_ and *u*_2_):

(8) $$\begin{array}{l} 0\leq u_{i}\leq \frac{g}{R_{\min}}. \end{array}$$

The workspace of a growing robot can now be described by evolving Equation (6) and imposing the constraint (8). Formally, there is always a path from any two points in 3D space (free of obstacles) that the robot can perform, with a desired destination and orientation; the only limiting factor on the workspace, when only geometric parameters are considered, is basically imposed by the material available to grow.

### Path Planning

**Figure 2:**
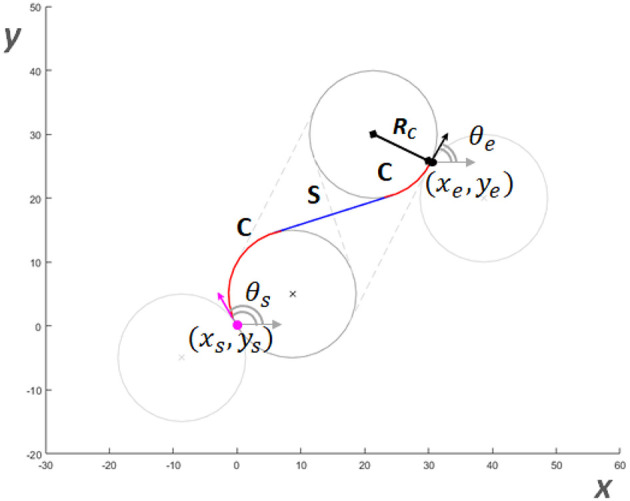
An example of Dubin's path in 2D, from a starting configuration in position **(*x***_***s***_**,*y***_***s***_**)** and orientation **θ**_***s***_ respect to the x-axis, to a target configuration having position in **(*x***_***e***_**,*y***_***e***_**)** and orientation **θ**_***e***_. The minimum path identified is composed by a first right curve, a straight line and a left curve. Each curvature has minimum curvature radius ***R***_***c***_.

Let's define an initial state $X_{s}=\langle x_{s},y_{s},z_{s},\theta_{s},\gamma_{s}\rangle$ and a target final state $X_{e}=\langle x_{e},y_{e},z_{e},\theta_{e},\gamma_{e}\rangle$ in the inertial frame. To find feasible paths for growing robots, solutions based on Dubin's path generation can be adopted bringing the tip from $X_{s}$ to $X_{e}$. Dubin's paths have been formalized for 2D motion planning of mobile robots and used to find optimal paths under curvature constraints (Dubins, 1957). In 2D, a minimum path is a path between a starting $Y_{s}=\langle x_{s},y_{s},\theta_{s}\rangle$ and a final state $Y_{e}=\langle x_{e},y_{e},\theta_{e}\rangle$ which can be composed by S straight segments or C curvatures, with several combinations: S, C, SC, CS, CSC, CC, CCC. If a solution with one or two segments is not available, the approach is to trace the tangent lines common to the four circles having $Y_{s}$ and $Y_{e}$ as tangent vectors, and selecting the path with minimum length (Figure 2). This approach guarantees the optimal solutions connecting $Y_{s}$ to $Y_{e}$. When moving the problem from 2D to 3D space, for instance to define the trajectory of unmanned aerial vehicles, the resolution of the minimum path becomes complex and computationally burdensome. For this reason, a suboptimal path by merging multiple approaches [e.g., Dubin's path, trajectory smoothing, interpolation between waypoints (Hwangbo et al., 2007; Ambrosino et al., 2009; Babaei and Mortazavi, 2010; Yu et al., 2015)] is typically proposed.

**Figure 3:**
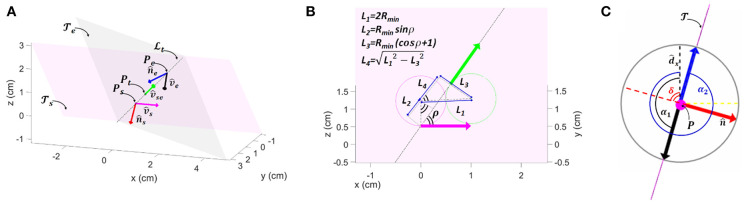
Schematic representation for Dubin's path approach used for 3D resolution. **(A)** The two planes $T_{\text{s}}$ and $T_{\text{e}}$ where starting ${\hat{v}}_{s}$ and target ${\hat{v}}_{e}$ vectors lie on. **(B)** Key parameters to localize the target position along the line of intersection between $T_{\text{s}}$ and $T_{\text{e}}$. **(C)** Top view of growing robot rip, with the two angles **α**_**1**_ and **α**_**2**_ of the possible deposition allowing the robot to move over the plane T. In **(C)** Dashed yellow line is the ***y***-axis of the tip coordinate system, black dashed line is the ***x***-axis and magenta is the ***z***-axis, where also the tip direction lies; blue and black arrows are the vectors toward the two possible angles of deposition.

Similarly, we addressed the problem of finding a suboptimal solution in 3D by dividing the problem into two optimal problems with curvature constraints: find the optimal path in 2D over two selected planes, $T_{s}$ and $T_{e}$, that intersect each other (where ${P_{s}=\left[x_{s}\;y_{s}\;z_{s}\right]}^{T}$ and ${P_{e}=\left[x_{e}\;y_{e}\;z_{e}\right]}^{T}$ lie, respectively) (Figure 3A).

We adopted a similar approach to Babaei and Mortazavi (2010), in which a trajectory is traced from a starting position that lies on one plane to a target position lying on a second plane, and passing from a waypoint located at the intersection of the two planes. In fact, if the tip of the growing robot arrives to lie on the intersecting line, it can easily pass from one plane to another just changing the deposition point (its α angle).

To select $T_{s}$ and $T_{e}$ we traced the line $L_{t}$ (Figure 3A) passing through *P*_*s*_ and *P*_*e*_, and we defined $T_{s}$ as the plane having the normal:

(9) $$\begin{array}{l} {\hat{n}}_{s}=\frac{{\hat{v}}_{se}\times {\hat{v}}_{s}}{{\left\|{\hat{v}}_{se}\times {\hat{v}}_{s}\right\|}_{2}}, \end{array}$$

and analogously, $T_{e}$ is selected as having the normal:

(10) $$\begin{array}{l} {\hat{n}}_{e}=\frac{{\hat{v}}_{se}\times {\hat{v}}_{e}}{{\left\|{\hat{v}}_{se}\times {\hat{v}}_{e}\right\|}_{2}}, \end{array}$$

where ${\hat{v}}_{s}$ is the unit vector of the tip direction at starting position, ${\hat{v}}_{e}$ is the unit vector of the tip direction at final position, and ${\hat{v}}_{se}$ is obtained by:

(11) $$\begin{array}{l} {\hat{v}}_{se}=\frac{P_{e}-P_{s}}{{\left\|P_{e}-P_{s}\right\|}_{2}}. \end{array}$$

This way, $L_{t}$ is also the intersecting line between $T_{s}$ and $T_{e}$. Over this line we should now identify a waypoint *P*_*t*_ which will be target position over plane $T_{s}$, as well as starting position over plane $T_{e}$; whereas the orientation of the tip is defined by ${\hat{v}}_{se}$. A valid point *P*_*t*_ should not be too much close to *P*_*s*_; this closeness can be defined by geometric constraints imposed by *R*_min_. To respect this constrain, we can define *P*_*t*_ as (Figure 3B):

(12) $$P_{t}=P_{s}+{\hat{v}}_{se}y^{*},$$

(13) $$\begin{array}{l} y^{*}=R_{\min}\sin\rho +\sqrt{{\left(2R_{\min}\right)}^{2}-{\left(R_{\min}\cos\rho +R_{\min}\right)}^{2}}+\epsilon . \end{array}$$

(14) $$\rho =\cos^{-1}\frac{{\hat{v}}_{s}\cdot {\hat{v}}_{se}}{{\left\|{\hat{v}}_{s}\right\|}_{2}{\left\|{\hat{v}}_{se}\right\|}_{2}}.$$

In (13), the ϵ is a small quantity (which can ϵ → 0) introduced just to overcome possible numerical approximation errors.

From now, the problem is divided in two 2D problems. We take the projection of *P*_*s*_ and *P*_*t*_ on the new reference system defined on plane $T_{s}$ (17) by extracting the first two components from, respectively vector *A* and *B* (${\left[a_{x}\;a_{y}\right]}^{-1}$ and ${\left[b_{x}\;b_{y}\right]}^{-1}$), which are obtained from the transformation:

(15) $$\begin{array}{l} {M_{s}}^{-1}P_{s}=A, \end{array}$$

(16) $$\begin{array}{l} {M_{s}}^{-1}P_{t}=B, \end{array}$$

(17) $$M_{s}=\left[\begin{array}{c} \begin{array}{cc} \begin{array}{cc} {\hat{v}}_{s} & {\hat{y}}_{s} \end{array} & \begin{array}{cc} {\hat{n}}_{s} & P_{s} \end{array} \end{array}\\ \begin{array}{cc} \begin{array}{cc} 0\; & 0\; \end{array} & \begin{array}{cc} 0 & \;1 \end{array} \end{array} \end{array}\right].$$

Vector *ŷ*_*s*_ is obtained as the orthonormal vector between ${\hat{n}}_{s}$ and ${\hat{v}}_{s}$:

(18) $$\begin{array}{l} {\hat{y}}_{s}=\frac{{\hat{n}}_{s}\times {\hat{v}}_{s}}{{\left\|{\hat{n}}_{s}\times {\hat{v}}_{s}\right\|}_{2}}, \end{array}$$

Analogously, to obtain the 2D coordinates of *P*_*t*_ and *P*_*e*_ on the reference system defined on plane $T_{e}$ (21), we extract the first two components of vector *C* and *D* (${\left[c_{x}\;c_{y}\right]}^{-1}$ and ${\left[d_{x}\;d_{y}\right]}^{-\;1}$):

(19) $$\begin{array}{l} {M_{e}}^{-1}P_{t}=C, \end{array}$$

(20) $$\begin{array}{l} {M_{e}}^{-1}P_{e}=D, \end{array}$$

(21) $$M_{e}=\left[\begin{array}{c} \begin{array}{cc} \begin{array}{cc} {\hat{v}}_{se} & {\hat{y}}_{e} \end{array} & \begin{array}{cc} {\hat{n}}_{e} & P_{t} \end{array} \end{array}\\ \begin{array}{cc} \begin{array}{cc} 0\; & 0\; \end{array} & \begin{array}{cc} 0\; & 1 \end{array} \end{array} \end{array}\right],$$

(22) $$\begin{array}{l} {\hat{y}}_{e}=\frac{{\hat{n}}_{e}\times {\hat{v}}_{se}}{{\left\|{\hat{n}}_{e}\times {\hat{v}}_{se}\right\|}_{2}}. \end{array}$$

By definition Equations (17) and (21), we have the heading angles of starting poses equal to 0, while we can obtain the heading angles for the target poses as:

(23) $$\theta_{s}=\cos^{-1}\frac{{\hat{v}}_{s}\cdot {\hat{v}}_{se}}{{\left\|{\hat{v}}_{s}\right\|}_{2}{\left\|{\hat{v}}_{se}\right\|}_{2}},$$

(24) $$\theta_{e}=\cos^{-1}\frac{{\hat{v}}_{e}\cdot {\hat{v}}_{se}}{{\left\|{\hat{v}}_{e}\right\|}_{2}{\left\|{\hat{v}}_{se}\right\|}_{2}}.$$

Therefore, the parameters of the minimum path problem on $T_{s}$ are provided by the initial state ${Y_{s}}^{T_{s}}=\langle a_{x},a_{y},0\rangle$ and the final state ${Y_{e}}^{T_{s}}=\langle b_{x},b_{y},\theta_{s}\rangle$, while for $T_{e}$ the parameters are ${Y_{s}}^{T_{e}}=\langle c_{x},c_{y},0\rangle$, and ${Y_{e}}^{T_{e}}=\langle d_{x},d_{y},\theta_{e}\;\rangle$.

Once we get the sequence of path segments, we can identify for each segment the action represented by the triple 〈α, β, *S*〉, where β = $\int_{\phi dt}$ is the angle representing the arc of the circle to be performed, $S=\int_{gdt}$ is the segment length, and α is the angle opposite to the curvature, on the *x-y* plane of the robot, which will indicate the point of deposition. α is found by first evaluating the angle δ between the plane where the robot is supposed to move (with normal $\hat{n}$), with the robot's *x* axis unit vector (${\hat{d}}_{x}$):

(25) $$\delta =\cos^{-1}\frac{\hat{n}\cdot {\hat{d}}_{x}}{{\left\|\hat{n}\right\|}_{2}{\left\|{\hat{d}}_{x}\right\|}_{2}},$$

then defining the two possible deposition angles, which should lie on the perpendicular line respect to $\hat{n}$, as $\alpha_{1}=\frac{\pi }{2}+\delta$ and $\alpha_{2}=\frac{3}{2}\pi +\delta$; and finally picking as α for each segment of the path, the one among α_1_ and α_2_ that is on the opposite direction of the projection of the *v*_*p*_ vector (the next waypoint in the sequence to be reached) on the *x-y* plane of the tip (*v*_α_ · *v*_*p*_*xy*__ = 1) (Figure 3C).

The triple 〈α, β, *S*〉 can thus be used as input parameter for Equation (4) to obtain ^*i*^*T* for each of the segment, and by (5) we get the kinematic chain of the robot from $X_{s}$ to $X_{e}$.

## Results

### Model Evaluation Over Different Distances of the Target Configuration

To evaluate the proposed kinematics we simulated the growth of a robot, implementing the equations of section Methods in MATLAB. We parametrized the simulations to fit the physical parameters of the growing robot implemented and deeply presented in Sadeghi et al. (2017) and Del Dottore et al. (2018c). The robot has an internal radius *r*_*t*_ of 2.2 cm, and an internal module with *L* equal to 4.8 cm and *r*_*r*_ of 1.2 cm, resulting in an *R*_min_ of 9.82 cm. This geometric evaluation agrees with the experiments performed on the robot and presented in Del Dottore et al. (2018c), in which we found a maximal deposition angle of 0.45° for a single layer having a maximal height of 0.095 ± 0.002 cm, thus resulting in an *R*_min_ = 9.79 cm. In the current work, we imposed _*R*_*c*_ = 10 *cm* ≥ *R*min_ in all our simulations to find the path from starting $X_{s}$ to the target configuration $X_{e}$. Moreover, our robot is able to deposit a single layer of material in 18 s and consequently for the simulated robot ϕ is equal to 0.025/*s* and the growing velocity is *g* = 0.0043 cm/s.

**Figure 4:**
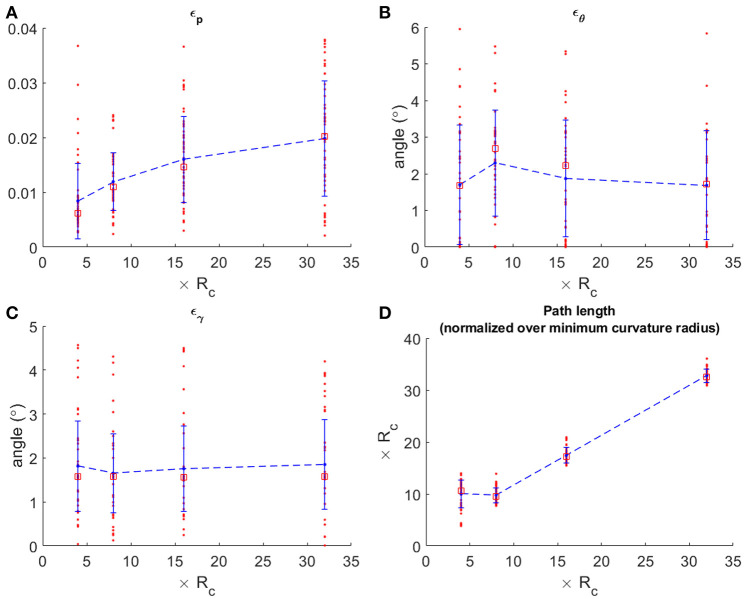
Performance achieved with the plant-inspired growing robot (Sadeghi et al., 2017) in simulation. **(A)** positional error over four groups of simulation 50 repetitions each, having random starting and target position with Euclidean distance **4*R***_***c***_, **8*R***_***c***_, **16*R***_***c***_, and **32*R***_***c***_; **(B,C)** orientation errors, heading and pitch, respectively; **(D)** final path length of the random paths that have been performed, normalized over the minimum curvature radius. In each graph, the red dots are the single simulation and red squares are median values. Mean values are connected by the dashed blue lines.

Four different groups of simulations were performed, with 50 repetitions each. The groups were composed setting the Euclidean distance between starting and target position (||_*P*_*s*_ − *Pe*_||_2_) of, respectively 4, 8, 16, and 32 times *R*_*c*_, and choosing for each repetition a completely random starting $X_{s}$ and target $X_{e}$ pose. At the end of each simulation we evaluated the error in position (ε_*p*_) as the distance between target position (*P*_*e*_) and the simulated robotic tip position (*T*_*e*_), normalized over the distance of the Dubin's path (*l*):

(26) $$\begin{array}{l} \varepsilon_{p}=\frac{{\left\|T_{e}-P_{e}\right\|}_{2}}{l}, \end{array}$$

and the errors in the heading (ε_θ_) and pitch angles (ε_γ_) as the distance between the target and achieved angles:

(27) $$\begin{array}{l} \varepsilon_{\theta }=|\theta_{t}-\theta_{e}|, \end{array}$$

(28) $$\begin{array}{l} \varepsilon_{\gamma }=|\gamma_{t}-\gamma_{e}|, \end{array}$$

where θ_*t*_ and γ_*t*_ are, respectively the heading and pitch angle achieved by the simulated robotic tip. The normalized error in position seems to slightly grow with the distance (Figure 4A) with a mean ranging from a lower value of 0.0084 ± 0.0069 to a maximum of 0.0198 ± 0.0105. The errors on heading and pitch are instead not affected by the distance, mean rank of each group is not significantly different from the others (*p*-value for heading error is 0.4327, and for pitch error is 0.6420), thus we can estimate a mean heading error of 1.89° ± 0.28° (Figure 4B) and a mean pitch error of 1.77° ± 0.09° (Figure 4C). Also, in a short path (≤ 4*R*_*c*_), the system due to its mechanical constraints is forced to travel a distance typically greater than the distance between starting and target pose, while in longer paths (> 4*R*_*c*_) the distance traveled almost resemble the distance between *P*_*s*_ and *P*_*e*_ (Figure 4D). Videos showing a schematic representation of the kinematic and examples of robot growth evolution in simulation from $X_{s}$ to $X_{e}$ are available as Supplementary Videos 1, 2. The error is mainly due to the discrete process of deposition, which induces an error between the desired waypoint (in the sequence of Dubin's path) and the actual position reached. Due to the small amount of material added at each step (in our case less than 1 mm) and the small angle (0.45°), the positional error remains relatively low.

### Contribution of Noise to the Model Error

**Figure 5:**
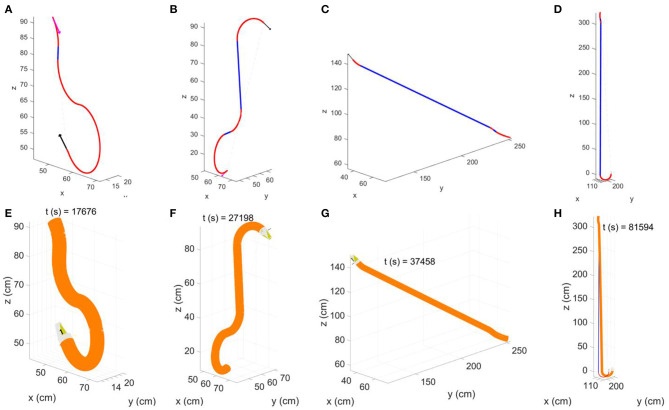
Representative paths extracted from each of the four groups (paths having the positional error close to the median error value). First row shows the Dubins' path obtained by the proposed path planner, while the second row shows the final configuration reached by the simulated robot with the corresponding time required to grow. **(A,E)** configuration having starting and target position with Euclidean distance of **4*R***_***c***_; **(B,F)** of **8*R***_***c***_; **(C,G)** of **16*R***_***c***_, and **(D,H)** of **32*R***_***c***_. In the Dubin's paths, blue segments are straight lines and red segments are curvilinear paths; the starting tip position and orientation is described by magenta arrow, and target position and orientation is described by the black arrow. The output of the simulation is described in the figure by the final robot body (orange) and tip (white and yellow) configuration.

**Figure 6:**
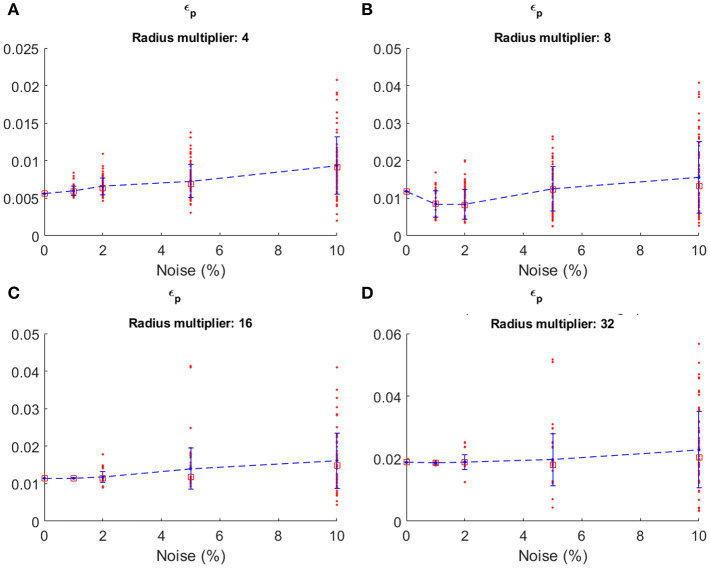
Positional error obtained with four different level of noise by the plant-inspired growing robot (Sadeghi et al., 2017) in simulation, over the four different groups of path: **(A) 4*R***_***c***_, **(B) 8*R***_***c***_, **(C) 16*R***_***c***_ and **(D) 32*R***_***c***_. In each graph, the red dots are the single simulation and red squares are median values. Mean values are connected by the dashed blue lines. The errors at 0 noise are the one obtained by the path closest to the median error from the previous set of simulations with no noise.

To verify the accuracy of the model, we introduced a random noise component to perturb the system. From each of the previously obtained group of simulations (4*R*_*c*_, 8*R*_*c*_, 16*R*_*c*_, 32*R*_*c*_) we extracted the path having ε_*p*_ closer to the median value of its group (Figure 5). For each of the selected path, we simulated the growth of the robot testing ±1, ±2, ±5, and ±10% of noise, calculated as a percentage of the averaged growth rate *g*, and used as additive noise to *g* at each time step. We limited the analysis to ±10% since, in the real system, we do not expect an excessively high noise in the growth rate. In fact, from previous experiments on the robot growing straight (Del Dottore et al., 2018c) we could find on average an error of ±2% in filament deposition height. We performed 70 repetitions for each level of noise in each group. Results (Figure 6) show stronger effects of the noise over short distances [increasing values - in 4*R*_*c*_ (Figure 6A) -or irregular trends - in 8*R*_*c*_ (Figure 6B)] rather than over long distance traveled (Figures 6C,D) (the one-way ANOVA test is reported in section Statistical analysis of the noise effects on the positional error of Supplementary Material).

### Effects of Robot Parameters Variation: Dimensions and Velocity

**Figure 7:**
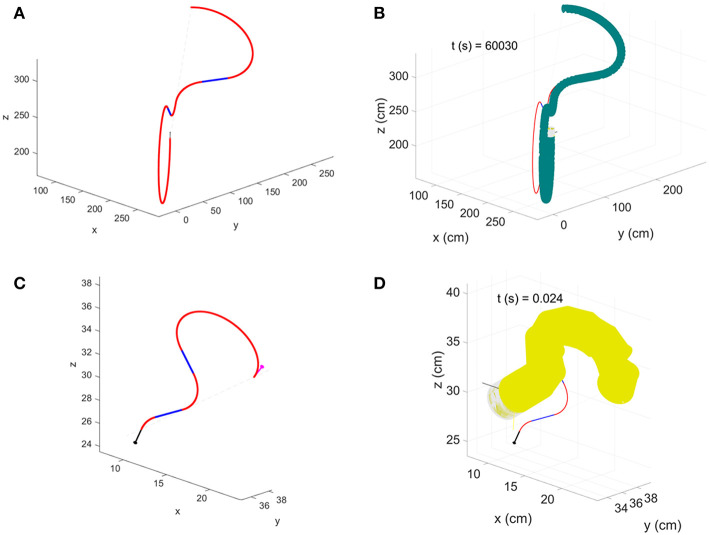
Paths having the positional error closer to the median value for the group of simulations having **4*R***_***c***_ as Euclidean distance between starting and target position. **(A)** Robot B Dubin's path and **(B)** configuration achieved at the end of simulation; **(C)** robot C Dubin's path and **(D)** configuration achieved at the end of simulation.

Additionally, we varied robot parameters to verify how robot dimensions and speed could affect model accuracy. Robot dimensions come into play in the kinematic model in the form of curvature radius ($R_{c}=\frac{g}{\phi }$ see Equations 4 and 7); thus, to analyze the behavior of the error, we created 8 different combination of *g* and *R*_*c*_, preserving a constant number of deposition over the same displacement, by setting constant *g* · *t* = 0.3096, where *t* is the time of material deposition for a single atomic step of growth (Table 1). To compare the performance among different robots, we calculated the index $k=\frac{g\cdot t}{R_{c}}$, which defines a ratio between robot minimum growth and its curvature radius. As before, we performed 4 different groups of path with 50 repetitions each having random $X_{s}$ and $X_{e}$. Positional errors with relative standard deviation are reported in Table 2. Ultimately, we looked at the error behavior emerging from robots having different time of deposition *t*. We set the parameters taking inspiration from the robots presented in Kayser et al. (2019) (named as robot B in the following) and Hawkes et al. (2017) (named as robot C), missing data have been estimated from the available information (Table 3). Also in this case, we performed four groups of simulations with 50 repetitions each, having random $X_{s}$ and $X_{e}$. Two examples of the paths performed by the simulated robot B and C are in Figure 7.

**Table 1:** Different parameterization of robot speed, curvature radius, and maximal intensity of bending ($\phi =\frac{g}{R_{c}}$).

	**Δ*t* (s)**	***g* (cm/s)**	***R*_*c*_ (cm)**	****ϕ** (rad/s)**	***k***
Robot A	18	0.0043	10	0.0004	0.0077
Robot A1	1.8	0.0430	10	0.0043	0.0077
Robot A2	0.18	0.4300	10	0.0430	0.0077
Robot A3	18	0.0043	30	0.0001	0.0026
Robot A4	1.8	0.0430	30	0.0014	0.0026
Robot A5	0.18	0.4300	30	0.0143	0.0026
Robot A6	18	0.0043	3.33	0.0013	0.0232
Robot A7	1.8	0.0430	3.33	0.0129	0.0232
Robot A8	0.18	0.4300	3.33	0.1290	0.0232

*Δ**t** represents the minimum time required by the robot for the minimum atomic step of growth. **k** is then defined as $\frac{\text{g}\cdot \Delta \text{t}}{{\text{R}}_{\text{c}}}$. All parameters for robot A have been extracted from the robot presented in Sadeghi et al. (2017); Del Dottore et al. (2018c)*.

**Table 2:** Mean positional error (±SD) achieved by each robot parameterization, over the four groups of path, with 50 repetition each.

	***4R*_*c*_**	***8R*_*c*_**	***16R*_*c*_**	***32R*_*c*_**
Robot A	0.0084 ± 0.0069	0.0119 ± 0.0052	0.0160 ± 0.0079	0.0198 ± 0.0105
Robot A1	0.0086 ± 0.0045	0.0135 ± 0.0065	0.0150 ± 0.0087	0.0179 ± 0.0106
Robot A2	0.0089 ± 0.0063	0.0126 ± 0.0064	0.0129 ± 0.0058	0.0164 ± 0.0110
Robot A3	0.0031 ± 0.0019	0.0025 ± 0.0010	0.0038 ± 0.0022	0.0059 ± 0.0028
Robot A4	0.0026 ± 0.0013	0.0031 ± 0.0028	0.0042 ± 0.0026	0.0059 ± 0.0039
Robot A5	0.0031 ± 0.0016	0.0031 ± 0.0021	0.0045 ± 0.0026	0.0059 ± 0.0040
Robot A6	0.0223 ± 0.0152	0.0411 ± 0.0181	0.0459 ± 0.0243	0.0445 ± 0.0223
Robot A7	0.0250 ± 0.0156	0.0557 ± 0.0427	0.0472 ± 0.0397	0.0440 ± 0.0332
Robot A8	0.0291 ± 0.0224	0.0370 ± 0.0183	0.0447 ± 0.0290	0.0399 ± 0.0277

**Table 3:** Parameters adopted for simulating the growth of robots having different size and growth velocity.

	**Robot A**	**Robot B**	**Robot C**
Δ***t***	18 s	522 s	0.0020 s
***g***	0.0043 cm/s	0.012 cm/s	1000 cm/s
***r***_***t***_	2.2 cm	5.75 cm	1.9 cm
***R***_***c***_	10 cm	68 cm	3.8 cm
**ϕ**	0.0004 rad/s	0.01 rad/s	4.47 rad/s
***k***	0.0077	0.0921	0.5263

*All parameters for robot A have been extracted from the robot presented in Sadeghi et al. (2017), Del Dottore et al. (2018c). Time of deposition **Δt**, the growth velocity **g**, the robot head radius **r**_**t**_ and the curvature radius **R**_**c**_ of robot B have been extracted from (Kayser et al., 2019), in which the robot was able to build 6.25 cm of structure in 8.7 min. From Hawkes et al. (2017) we extracted robot head dimension **r**_**t**_ and the growth velocity **g** for robot C. **Δt** has been obtained assuming to have 1 latch every 1 cm and alternated on opposite sides of the robot (on the same side, 2 consecutive latches have a distance of 2 cm). When pressurized, a latch releases 2 cm of material. Thus, for a single step of bending, robot C will have **h**_**1**_ = 1 cm of material on one side and **h**_**2**_ = 3 cm on the opposite side. In this case we evaluated $R_{c}=r_{t}\frac{h_{1}+h_{2}}{h_{2}-h_{1}},\Delta t=\frac{h_{1}+h_{2}}{2}\frac{1}{g}$ and $\phi =\frac{h_{2}-\;h_{1}}{2r_{t}}$*.

**Figure 8:**
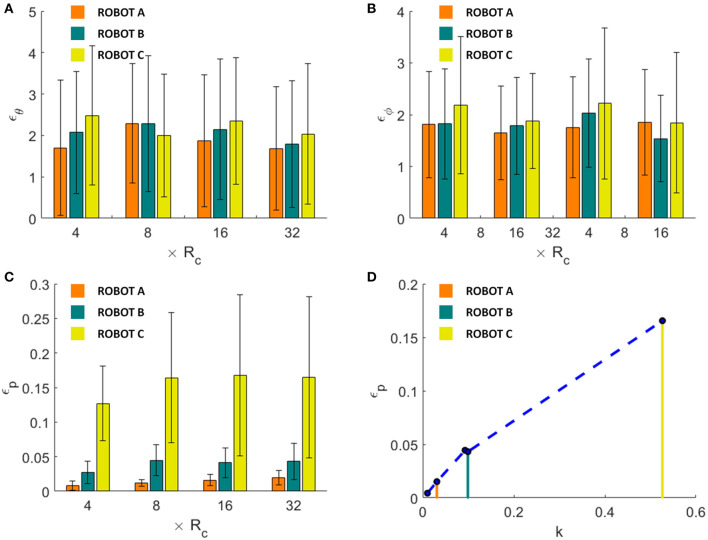
Comparison of mean heading **(A)**, pitch **(B)** and positional **(C)** errors achieved by robot A, B and C for each of the simulation groups. In **(D)**, the positional error is shown as a function of the ratio ***k*** between discretization step (***g* · *t***) and curvature radius ***R***_***c***_. The dots in graph B represent the mean positional errors achieved by the simulations with the parameters as presented in Tables 1, 3, averaged among groups **8*R***_***c***_, **16*R***_***c***_, and **32*R***_***c***_. Results obtained by robot are highlighted by the corresponding colored lines. Performances of robot A in all the graphs refer to the results as shown in Figure 4, but are here reported for easiness of comparison.

Results demonstrate that heading and pitch errors are unaffected by variation of parameters, showing a not significantly different behavior among all the simulations obtained with parameters as in Tables 1, 3 (*p*-value for heading error is 0.5578, and for pitch error is 0.1490) with an average error of ~2° ε_θ_ (Figure 8A) and ~1.8° ε_γ_ (Figure 8B). Whereas, ε_*p*_ tends to stabilize in long distances (path length ≥ 8*R*_*c*_) in robot B and C (*p*-value in long paths with robot B is 0.7719, and with robot C is 0.9160), reaching a mean error of 0.0043 ± 0.0023 with robot B and of 0.1655 ± 0.1089 with robot C (Figure 8C). Moreover, for constant *k* the error ε_*p*_ is not affected (the one-way ANOVA test is reported in section Statistical analysis to evaluate the significance of different parameterization of Supplementary Material), while it increases with *k* (Figure 8D). Conditions for high *k* values are small curvature radius or large discretization step (*g* · *t* = robot B 6.26 cm ≫ robot C 2 cm ≫ robot A 0.077 cm) (Table 3) which induces to accumulate errors in reaching each sequence target position and amplifies this effect in tight curvilinear paths (case of robot C with *R*_*c*_ = 3.8 cm).

## Discussions

The motion obtained by growing from the tip is becoming an attractive ability in robotics since it can enable robots to navigate their environments by adapting their bodies and morphologies to the constraints of the surrounding. The body is built in real-time by the robot, according to environmental and task demand, through the addition of new material at the tip, driving in this way the tip navigation. This means that the robot's path is not predictable *a priori*.

Navigation of unstructured environments cannot rely on classic map-based path planning strategies; the robot in those cases should move with a higher level behavior control, i.e., a stimuli-oriented control (Sadeghi et al., 2016). In this context, a perfect knowledge of the robot kinematic is fundamental for understanding the feasibility of the path chosen by the behavioral control. The proposed kinematic model can be adopted, coupled with the higher control, to help in localizing the robot or to predict its next position. Moreover, the proposed kinematic control can be used in short-distance navigation: when for instance the robot has the possibility to reconstruct the close surrounding by means of its own perception (e.g., vision, tactile, depth sensors). In this view, the robot can set a proximal waypoint, define its path, and reach the target.

The key parameter defining the path and the ability of a growing robot to adapt through different unknown patterns is the minimum curvature radius. This parameter is affected, and consequently, the space of maneuverability may be limited, by the design of the robot and particularly by the size of mechanical components (e.g. motors, other actuators, and components). Here, a parameterization of the growing system mechanical design is presented and formulation of the curvature radius in terms of that parameters proposed, giving a good agreement with experimental results, i.e., we geometrically evaluated the minimum curvature radius of our growing robot as *R*_min_ = 9.82 cm, whereas by previous experiments we found *R*_min_ = 9.79 (Del Dottore et al., 2018c).

Yet, our analysis has been limited to a geometric evaluation aimed at characterizing the motion of growing from the tip robots. By looking at the kinematics, we evaluated the theoretical workspace of growing robots, however, when deepening in the analysis of the motion, dynamics of each specific system should be also considered. For instance, when a growing robot moves in the air, the weight of the tip and the suspended part of the built body should be carefully taken into account in the control dynamics, in order to prevent the structural collapse. In fact, speed and forces acting on a robotic system play a relevant role which could address the features of the robot from one application to another. Also, when designing the robot, the selection of the growth mechanism is particularly important when talking about applications. For instance, for biomedical applications, in the design of a growing robot, the reversibility of the system and the biocompatibility of the growth strategy and building material are fundamental, whereas, in a rescue scenario, the speed and robustness become much more relevant.

## Conclusions

This paper formalizes the kinematics model for growing robots, setting the analogy with mobile non-holonomic systems, and shows the ability of the model to describe the motion of a plant-inspired growing robot. Given a starting and a final pose in the 3D space, here we defined a kinematic control to connect them. We propose to split the global movement into two optimal planar paths based on Dubin's solution and we formalize our approach finding the two planes and the trajectories above them. We verified our strategy with different poses in simulation demonstrating the ability of a plant-inspired growing robot to reach the expected final position with the desired orientation (maximal positional error of ~6 cm in 320 cm of path length and ~1.8° in orientation errors). We also evaluated the effects of different level of noise, and the effects of different model parametrization. We noted that not only the curvature radius but also the specific discretization of the robot affect its ability in reaching, with high or low accuracy, the desired point and thus must be taken into account when defining a feasible path. However, our analysis generally shows the accuracy of the proposed strategy, when considering an almost continuous growth of the robot, the efficacy of the model and its applicability over different sizes, curvature radius, and growth speeds.

However, when moving from simulation to physical implementation, the kinematic analysis is not enough to correctly analyze robot motion. Future steps will focus on formalizing the optimal path considering specific characteristics of the robot into the model, particularly, evaluating how the dynamics (considering self-weight and other forces exercised in interaction with the environment during growth) would affect the path, and implementing the strategy on the robo-physical model (Sadeghi et al., 2017). Additionally, positional and orientation errors between simulation and a real robot would be considered and corrected, at least partially, by adopting internal odometry sensors and inertial measurement units, which would allow inserting feedback about the actual material deposition into a closed-loop control.

## Author Contributions

ED conceived and formalized the model. ED and AM discussed model and experiments. ED, AM, AS, and BM discussed results and wrote the paper.

### Conflict of Interest Statement

The authors declare that the research was conducted in the absence of any commercial or financial relationships that could be construed as a potential conflict of interest
