# Supplementary material for: Characterization of the Growing From the Tip as Robot Locomotion Strategy
Source: Front Robot AI. 2019 Jun 20;6:45. doi: 10.3389/frobt.2019.00045 (PMC7805678; doi:10.3389/frobt.2019.00045)
Supplement: Supplementary file 3 [file Data_Sheet_1.pdf]

Supplementary Material

Characterization of the Growing from the Tip as Robot Locomotion Strategy

Emanuela Del Dottore[1*], Alessio Mondini[1], Ali Sadeghi[1], Barbara Mazzolai[1*]

[1]Center for Micro-BioRobotics, Istituto Italiano di Tecnologia, Pontedera, Italy

*** Correspondence:**
Emanuela Del Dottore;
emanuela.deldottore@iit.it
Barbara Mazzolai
barbara.mazzolai@iit.it

1 Nomenclature

Symbol	Description
j	inertial frame
i	frame solid with robotic tip
${}^{j}p_i$	origin of coordinate system i relative to coordinate system j
${}^{j}x_i$ or x	component x of a point in coordinate system i relative to coordinate system j
${}^{j}y_i$ or y	component y of a point in coordinate system i relative to coordinate system j
${}^{j}z_i$ or z	component z of a point in coordinate system i relative to coordinate system j
${}^{i}r$	a generic point in coordinate system i
${}^{j}r$	a generic point in coordinate system j
${}^{j}R_i$	a transformation matrix from frame i to frame j
${}^{j}T_i$	an homogenous transformation matrix from frame i to frame j
P_d	plane of deposition in the robotic tip
g	growth velocity
α	angle of greatest material deposition with respect to the x-axis in frame i
ϕ	intensity of bending in a unit of time
R_c	curvature radius
ICR	inertial center of rotation
s_0	initial position of a trajectory
s_e	final position of a trajectory
s_t	a generic position along a trajectory
S	length of the trajectory from s_0 to s_e
t	time
θ	heading angle of the robotic tip
γ	pitch angle of the robotic tip
u_i	input control variables, for $i = 1,2$
R_{min}	minimum curvature radius
r_t	tip radius
r_r	maximum radius of internal components assembly
L	maximum length of internal components assembly
CP	contact point along the body structure of internal components with the internal side of the body

$\mathcal{X}_s$	initial state in 3D inertial frame, composed by position (x_s, y_s, z_s), heading (θ_s), and pitch (γ_s)
$\mathcal{X}_e$	final state in 3D inertial frame, composed by position (x_e, y_e, z_e), heading (θ_e), and pitch (γ_e)
$\mathcal{Y}_s$	initial state in 2D inertial frame, composed by position (x_s, y_s), and heading (θ_s)
$\mathcal{Y}_e$	initial state in 2D inertial frame, composed by position (x_e, y_e), and heading (θ_e)
$\mathcal{T}_s$	selected starting plane
$\mathcal{T}_e$	selected ending plane
P_s	starting point
P_e	ending point
$\mathcal{L}_t$	intersecting line between two planes
P_t	a waypoint along $\mathcal{L}_t$
$\hat{n}$	normal to a plane (with subscript s, plane $\mathcal{T}_s$, with subscript e, plane $\mathcal{T}_e$)
$\hat{v}$	unit vector of tip direction (if subscript s in plane $\mathcal{T}_s$; if subscript e in plane $\mathcal{T}_e$; if subscript se in line $\mathcal{L}_t$)
y^*	a minimum distance between starting point P_s and the waypoint along $\mathcal{L}_t$
ρ	angle between tip orientation and intersecting line
ϵ	an arbitrary small quantity
A, B, C, D	2D projection of four 3D points (respectively: P_s, P_t, P_t, P_e)
M_s	transformation matrix to convert 3D coordinates from plane $\mathcal{T}_s$ into 2D coordinates
M_e	transformation matrix to convert 3D coordinates from plane $\mathcal{T}_e$ into 2D coordinates
$\hat{y}_s$	orthonormal vector to $\hat{v}_s$ and $\hat{n}_s$
$\hat{y}_e$	orthonormal vector to $\hat{v}_e$ and $\hat{n}_e$
θ_s	heading angle of target pose in plane $\mathcal{T}_s$ in 2D
θ_e	heading angle of target pose in plane $\mathcal{T}_e$ in 2D
β	angle for the arc in the circle of the Dubin's path
δ	angle between the plane where the robot is supposed to move with the robot's *x* axis orientation
$\hat{d}_x$	the robot's *x* axis unit vector
α_1, α_2	the two possible angles of deposition allowing the tip to move in 2D plane
v_α	vector from tip coordinate system origin towards α-point
v_p	vector from tip coordinate system origin towards p-point, the next waypoint in the sequence to be reached
$v_{p_{xy}}$	projection of v_p on the *x*-*y* plane of the tip
ε_p	positional error
T_e	final position of the simulated robotic tip
l	Dubin's path length
θ_t	final heading angle of the simulated robotic tip
ε_θ	heading error
γ_t	final pitch angle of the simulated robotic tip
ε_γ	pitch error
Δt	time step of atomic material deposition
h_1	the height of the material deposited at the internal side of the curvature
h_2	the height of the material deposited at the external side of the curvature
k	evaluation index defining the ratio between robot minimum step of growth and its curvature radius

2 Statistical analysis of the noise effects on the positional error

All the simulations have been collected and grouped according to the distance that has been imposed between starting and target positions ($4R_c$, $8R_c$, $16R_c$, $32R_c$). Each group has five subsets with 70 repetitions each: 0 noise, ±1, ±2, ±5 and ±10% of noise. To evaluate if the resulting mean errors are significantly different among different level of noise, we performed the Kruskal-Wallis ANOVA test for each of the four groups. The p-value resulting from the analysis is reported in Table S1. In short distance ($4R_c$), the 0 noise group has a mean rank significantly different from the others, whereas, in the other level of noise, the significance between groups is strongly reduced (e.g. groups ±2%, ±5% and ±1%, ±2% are not independent) (Fig. S1). In the group of $8R_c$, the 0 noise is significantly

different only from group ±1% and ±2%, and in $16R_c$ with ±5% and ±10%. With $32R_c$ there are no groups having mean ranks significantly different from each other, considering 1% significance level.

3 Statistical analysis to evaluate the significance of different parameterization

Results of simulations among different parameterization (Table 1 in main text) have been compared by grouping them in three sets, according to the same k value: {A, A1, A2}, {A3, A4, A5} and {A6, A7, A8}. For each set, the *p*-value among the four groups of path has been calculated (Table S2). None of the groups showed mean ranks significantly different from the others (1% significance level).

Table S1: *p*-values resulting from the Kruskal-Wallis ANOVA test over the four groups of path simulations with noise.

	$4R_c$	$8R_c$	$16R_c$	$32R_c$
***p*-value**	$3.4728 \cdot 10^{-21}$	$4.9269 \cdot 10^{-11}$	$1.8477 \cdot 10^{-06}$	0.1032

Table S2: *p*-values resulting from the Kruskal-Wallis ANOVA test over the four groups of path simulations. Each row groups robots having same k value but different growth velocity.

	$4R_c$	$8R_c$	$16R_c$	$32R_c$
{A, A1, A2} ***p*-value**	0.1014	0.5478	0.1812	0.2253
{A3, A4, A5} ***p*-value**	0.2299	0.4865	0.3051	0.8518
{A6, A7, A8} ***p*-value**	0.6664	0.6568	0.8200	0.7818

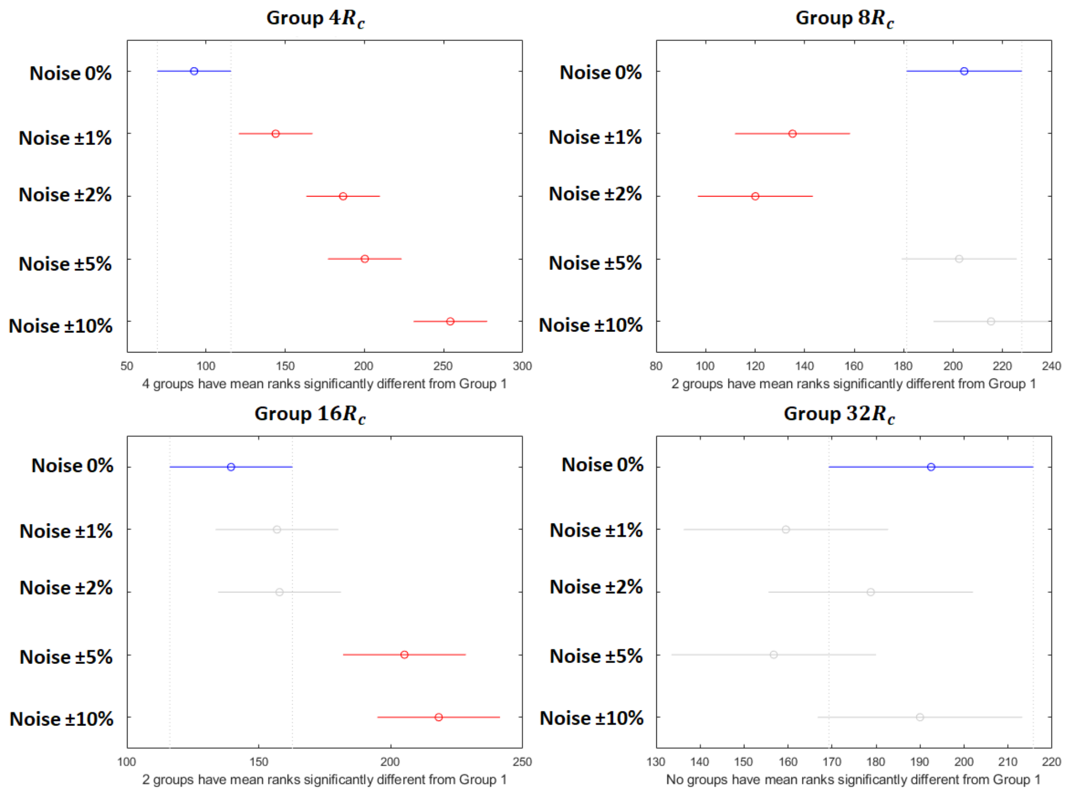

Figure S1: Box results of Kruskal-Wallis ANOVA test over the path simulations with noise presenting the significance among subsets for each of the group. Just the significance between the set performed with the 0 noise with the others is highlighted.
